# A study of ^18^F-FLT positron emission tomography/computed tomography imaging in cases of prefibrotic/early primary myelofibrosis and essential thrombocythemia

**DOI:** 10.1097/MD.0000000000023088

**Published:** 2020-11-06

**Authors:** Mohamed A. Yassin, Sadek A. Nehmeh, Abdulqadir J. Nashwan, Samah A. Kohla, Shehab F. Mohamed, Omar M. Ismail, Ahmad Al Sabbagh, Firyal Ibrahim, Dina S. Soliman, Lajos Szabados, Hadi Fayad

**Affiliations:** a Department of Medical Oncology/Hematology, National Centre for Cancer Care and Research, Hamad Medical Corporation, Doha, Qatar; b Department of Radiology, Weill Cornell Medical College, New York, NY; c Department of Education and Practice Development, Hazm Mebaireek General Hospital (HMGH), Hamad Medical Corporation; d Department of Laboratory Medicine and Pathology, Hematopathology Section, Hamad Medical Corporation; e Radiology Department, Hamad Medical Corporation; f Occupational Health and Safety Department, PET/CT Centre, Hamad Medical Corporation, Doha, Qatar.

**Keywords:** fluorothymidine F-18, positron emission tomography, essential thrombocythemia, prefibrotic/early primary myelofibrosis

## Abstract

The objectives of this research project are to study in patients with primary myelofibrosis (PMF) and Essential Thrombocythemia (ET); (1) the uptake patterns of ^18^FLT-PET (FLT-PET) and its value in diagnosing, staging, and treatment response monitoring of malignant hematopoiesis, (2) compare imaging findings from FLT-PET with bone marrow biopsy (standard of care), and (3) associate FLT-PET uptake patterns with genetic makeup such as JAK2 (Janus kinase 2), CALR (Calreticulin), MPL (myeloproliferative leukemia protein), Triple negative disease, and allele burden.

This trial is registered in ClinicalTrials.gov with number NCT03116542. Protocol version: Mar 2017

## 1 Introduction

Positron emission tomography (PET) with fluorodeoxyglucose (^18^F-FDG) combined with computed tomography is a major tool for the diagnosis, staging, and monitoring of treatment response in clinical oncology.^[1]^ 3′-18Fluoro-3′-deoxy-L-thymidine (FLT) with PET is a surrogate for assessing noninvasively.^[2]^

Myeloproliferative Neoplasms (MPNs) are clonal hematopoietic stem disorders are characterized by high rate of effective proliferation of one or more cell lineage. MPNs exhibit overlapping syndromes that can progress to fibrotic stage or progress into acute leukemia. Preliminary results of a pilot study^[3]^ suggested that this technique (FLT-PET) could be useful to assess bone marrow (BM) activity and extramedullary hematopoiesis in patients with Myelofibrosis (MF).

The current standard of care approach for follow-up in patients with MF is based on pathological markers (peripheral blood counts and bone marrow histomorphology) and molecular markers. Bone marrow examination is considered as the gold standard diagnostic method in MF patients. It provides detailed information about cellularity, morphology of each lineage, the degree of fibrosis, and, transformation and dysplastic features. However, many patients are reluctant to go for this invasive technique which precludes precise assessment of disease activity at the desirable frequencies. Alternative noninvasive clinical techniques are still lacking.

In a recent study, Vercellino et al showed the feasibility of FLT-PET to evaluate the diagnostic value of this technique in in primary myelofibrosis where ^18^F-FLT PET appears to be a reliable and convenient technique to assess hematopoietic activity in bone marrow.^[4]^ Assessing the patterns of ^18^FLT uptake in the different subgroups of MPNs are still however needed. In this study protocol we propose to study and characterize FLT uptake patterns in PET images of early prefibrotic stages of PMF (pre-PMF) in different clinical and genetic settings, and Essential Thrombocythemia (ET).^[4]^ Specifically, we will assess the value of FLT-PET uptake patterns in diagnosis and treatment response assessment of PMF patients. We will also examine their feasibility to differentiate between early stage PMF and ET, and will correlate those with disease activity, degree of fibrosis, and early detection of acute transformation.

## 2 Objectives

The objective of this study is to:

- Assess the value of FLT-PET uptake patterns in the diagnosis, staging, and response to therapy monitoring of malignant hematopoiesis in patients with pre-PMF and ET diseases.
- Identify different patterns of uptake in patients with pre-PMF and ET in different clinical settings.
- Compare the values of FLT-PET in disease diagnosis, assessment of disease activity, detection of transformation, monitoring of treatment response, and grading of fibrosis to those of bone marrow biopsy (gold standard) in pre-PMF and ET.
- Study the ability of FLT-PET to differentiate between pre-PMF and ET.
- Correlate FLT-PET uptake patterns with different genetic makeup (JAK2, [calreticulin] CALR, MPL, or Triple negative disease) or allele burden in patients with pre-PMF and ET.

## 3 Trial design

This is a phase-I, Diagnostic, Single Group Assignment, Open Label clinical study

## 4 Methods: participants, interventions, and outcomes

### 4.1 Study setting

The study will take place in the National Center for Cancer Care and Research (NCCCR) exclusively where the hematology outpatient, hematology lab, and PET/CT departments will be involved. All patients are diagnosed and/or followed in NCCCR, and all the investigators on this protocol are from NCCCR. The details of the procedure and interventions are mentioned below in the background and methods sections.

### 4.2 Eligibility criteria

1. Cases fulfilling WHO2016 diagnostic criteria for PMF or ET.^[5]^
2. WHO criteria for pre-PMF.

#### 4.2.1 Major criteria

1. Megakaryocytic proliferation and atypia, without reticulin fibrosis > grade 1∗, accompanied by increased age-adjusted BM cellularity, granulocytic proliferation and often decreased erythropoiesis
2. Not meeting the WHO criteria for *BCR-ABL1*+ CML, PV, ET, myelodysplastic syndromes, or other myeloid neoplasms
3. Presence of *JAK2*, *CALR* or *MPL* mutation or in the absence of these mutations, presence of another clonal marker∗∗or absence of minor reactive BM reticulin fibrosis.

#### 4.2.2 Minor criteria:

Presence of at least one of the following, confirmed in 2 consecutive determinations:

1. Anemia not attributed to a comorbid condition
2. Leukocytosis >11 × 109/L
3. Palpable splenomegaly
4. LDH increased to above upper normal limit of institutional reference range.

### 4.3 Diagnosis of pre-PMF requires meeting all 3 major criteria, and at least one minor criterion

∗∗In the absence of any of the 3 major clonal mutations, the search for the most frequent accompanying mutations (e.g. *ASXL1, EZH2, TET2, IDH1/IDH2, SRSF2, SF3B1*) are of help in determining the clonal nature of the disease

**∗∗∗**Minor (grade 1) reticulin fibrosis secondary to infection, autoimmune disorder, or other chronic inflammatory conditions, hairy cell leukemia or other lymphoid neoplasm, metastatic malignancy, or toxic (chronic) myelopathies

WHO diagnostic criteria ET Major criteria Platelet count ≥450 × 109/L Bone marrow biopsy showing proliferation mainly of the megakaryocyte lineage with increased numbers of enlarged and mature megakaryocytes with hyperlobulated nuclei. No significant increase or left shift in neutrophil granulopoiesis or erythropoiesis and very rarely minor (grade 1) increase in reticulin fibers.

Not meeting WHO criteria for BCR-ABL1+ CML, PV, PMF, myelodysplastic syndromes, or other myeloid neoplasms. Presence of JAK2, CALR, or MPL mutation Minor criterion Presence of a clonal marker or absence of evidence for reactive thrombocytosis. Diagnosis of ET requires meeting all 4 major criteria or the first 3 major criteria and the minor criterion

### 4.4 Other inclusion criteria

- Age 18 years old or above.
- Patient accept to sign inform consent.
- ECOG performance less than or equal 2.

## 5 Exclusion criteria

1. Patient not fulfilling the inclusion criteria.
2. Vulnerable groups: pregnant, minors, and prisoners will be excluded.

Bone marrow will be collected as part of the routine diagnostic work-up. No extra bone marrow material will be collected solely for the aim of the study.

### 5.1 Outcomes

Primary outcome measure:

- Number of patients with ≥ 50% increase in FLT uptake in the PET images compared to and at 12 months post-baseline scan in patients with PMF and ET.
  ∘ Examine the value of FLT-PET in assessing malignant hematopoiesis in patients with PMF and ET [Time Frame: 12 Months]

Secondary outcome measures:

- Reduction of >50% of allele burden of different mutations (JAK-2, MPL, CALR) and its expression on FLT- PET uptake pattern 12 moths post-baseline scan.
  ∘ Correlation of different mutations (JAK-2, MPL, CALR) expression and allele burden with FLT- PET uptake patterns [Time Frame: 12 Months].

### 5.2 Participant timeline

Expected time of the trial will be 1 calendar year after ethical approval and will be renewed annually for 5 years.

### 5.3 Sample size and recruitment

All patients will undergo static whole-body FLT PET/CT imaging at baseline in a cohort of 21 patients.

## 6 Methods: data collection, management, and analysis

All enrolled patients will undergo a baseline FLT PET/CT at baseline. All PET data will be acquired on the Siemens mCT PET/CT scanner in 3D mode. FLT-PET scans will be performed in static mode 60 minutes ± 10 minutes postinjection.

Diagnostic agent [^18^F] FLT: FLT will be synthesized by the HMC Radiochemistry Facility. The target patient activity will be 10 mCi ± 10%. The product will be required to meet all of the release acceptance criteria outlined by HMC policies for purity, sterility, endotoxin, and identity before administration.

Patient preparation and administration of FLT-PET: No specific dietary restrictions or hydration are required for FLT-PET scans. However, patients will be urged to drink plenty of water before and after the PET studies. FLT will be prepared by the HMC cyclotron Core Facility and assessed for quality control following “good manufacturing practice” criteria. For each scan, patients will receive approximately up to 10 mCi ± 10% FLT by intravenous infusion.

Whole body scan:

The baseline FLT-PET study will be performed in static mode as follows: Static FLT-PET acquisition:

- Scout view and low dose CT (see below) for 2 to 3 PET FOV's (∼24 cm per FOV) will be obtained.
- Static PET acquisition will start at 60 min ± 10 min post-injection at 3 minutes per PET field-of-view.

All studies will be carried out on a Siemens mCT PET/CT scanner residing at the PET center at HMC, and in 3D mode.

Image analysis and interpretation details:

All FLT-PET images will be reviewed and examined by a board-certified physician who is a member of the HMC PET/CT or radiology services, and who is experienced in PET interpretation. CT, attenuation-corrected PET, and fused PET-CT images will be available and reviewed simultaneously for each patient. Interpretation of the cancer site will be based on lesion SUV, facility of radiotracer uptake, and detectability.

**Bone marrow examination**: Bone marrow biopsy will be performed as a part of routine work-up for all selected patients before the FLT-PET scan. Subjects will be categorized based on their diagnosis; ET/Prefibrotic myelofibrosis vs borderline undifferentiated, and ET vs/or Prefibrotic.

### 6.1 Data management

Clinical, pathological, radiological, and molecular data will be compiled in data excel sheet. The clinical, pathological, and genetic data will be correlated to the FLT-PET uptake patterns.

A senior PET/CT technologist who is already involved in research will be assigned to the study. The responsibilities of the designated person include project compliance, data collection, abstraction and entry, data reporting, regulatory monitoring, problem resolution and prioritization, and coordination of the activities of the protocol study team.

The data collected for this study will be transferred to a secure database managed by the HMC IT team (eg, PACS). All data generated in this study will be the property of HMC. Original data acquired on the PET/CT scanner console will be managed as per the PET/CT center policy and procedures regulations.

Source documentation will be available to support the computerized patient record. Study personnel will record clinical data in each patient's source documents (ie, the patient's medical record). The study team will maintain adequate and accurate records to enable the conduct of the study to be fully documented and the study data to be subsequently verified. After study closure, the investigators will maintain all source documents, study-related documents, and the data stored in the database used for data collection. Data will be entered throughout the duration of the trial as patients are enrolled.

Clinical, pathological, radiological, and molecular data will be compiled in data excel sheet. The clinical, pathological and genetic data will be correlated to uptake pattern of FLT-PET.

A senior PET/CT technologist who is already involved in research will be assigned to the study. The responsibilities of the designated person include project compliance, data collection, abstraction and entry, data reporting, regulatory monitoring, problem resolution and prioritization, and coordinate the activities of the protocol study team.

The data collected for this study will be transferred to a secure database managed by the HMC IT team (eg, PACS). All data generated in this study will be the property of HMC. Original data acquired on the PET/CT scanner console will be destroyed as per the PET/CT center policy and procedures regulations.

Source documentation will be available to support the computerized patient record. Study personnel will record clinical data in each patient's source documents (ie, the patient's medical record). The study team will maintain adequate and accurate records to enable the conduct of the study to be fully documented and the study data to be subsequently verified. After study closure, the investigator will maintain all source documents, study-related documents, and the data stored in the database used for data collection. Data will be entered throughout the duration of the trial as patients are enrolled.

### 6.2 Statistical methods

The primary aim is to study the ability of FLT-PET in differentiating between pre-PMF and ET. The focus of the data analysis in our study is to determine the predictive value and accuracy of FLT-PET uptake in predicting diagnosis. For this purpose, we will use receiver-operating-characteristic (ROC) curves and will compute areas under curves for each parameter for its robustness in predicting diagnosis. In addition, we will create a model probability cutoff graph to choose parameters that best predict response and determine cutoffs that showed highest accuracy (sensitivity, specificity, positive, and negative predictive values of these parameters). The ROC curves provide a comprehensive and visually attractive way to summarize the accuracy of predictions. The ROC curve shows the tradeoff between sensitivity and specificity and is a better method to detect the performance of a developed test. Kruskal-Wallis will be also used in addition or to replace ROC curves depending on the final number of recruited patients and statistical conditions. A 2-sided *P* value <0.05 will be considered to be statistically significant. All statistical analyses will be done using MedCalc (Medcalc Software, Ostend, Belgium).

## 7 Methods: monitoring

### 7.1 Data monitoring and auditing

All documents related to this clinical trial, including the informed consent signed by the patients will be securely stored and according to the JCI, MRC, and MoPH requirements. Any side effects or adverse events related to the trial will be reported to the hospital research committee and the medical research center.

Regular registration report will be generated to monitor patient accrual and completeness of registration data. Routine data quality report will be generated to assess missing data and inconsistencies. Accrual rate, extent and accuracy of evaluations, and follow-up will be monitored periodically throughout the study period, and potential problems will be brought to the attention of the study team for discussion and action. Random-template data quality and protocol compliance audits may be conducted by the study team, at a minimum of once per year, or more frequently if indicated.

Data safety and monitoring will be conducted according to the HMC-IRB, ethics, and data safety monitoring board regulations.

### 7.2 Toxicity of ^18^F-FLT

FLT has been used in over 300 patients worldwide and no side effects related to the radiotracer have been reported. In a study performed at the University of Washington, Turcotte and colleagues^[6]^ assessed the toxicity of [^18^F] FLT in 20 patients with proven or suspected diagnosis of non-small cell lung cancer. Blood samples were collected for each patient at multiple times before and after [^18^F] FLT-PET. These samples were assayed for comprehensive metabolic panel, total bilirubin, and, blood and platelet counts. In addition, a standard neurological examination by a qualified physician was performed for each patient before and immediately after [^18^F] FLT-PET. No side effects were reported by patients or observed. No change in the neurological status of the patients was observed either. In this clinical trial, patients will undergo a regular follow up in hematology clinic, and any adverse effect will be reported to the Hospital Research Committee (HRC) and the Medical Research Center (MRC) in Hamad Medical Corporation (HMC) by the Principal investigator.

Due to the trace amount of FLT injected, no allergic reactions or possible kidney damage will be expected.

#### 7.2.1 Radiation dose

The amount of injected activity determines the radiation dose from the PET part of the procedure. With proper hydration of the patient the excretion of the tracer can be forced further reducing the received dose. The radiation dose from the PET part is in the order of 5 to 10 mSv.

During PET/CT imaging, the CT part is used to improve PET image quality (attenuation correction) and for anatomic localization so quite low dose CT can be used without CT contrast injection. The effective dose from CT is also in the order of. Therefore, the effective dose from PET/CT procedure is in the order of 20 mSv per FLT dose.

## 8 Ethics and dissemination

### 8.1 Research ethics approval

The study is approved (full-board) by the IRB-Medical Research Center (MRC) at Hamad Medical Corporation (HMC) in Qatar (16287/16).

### 8.2 Informed consent

Subjects will be screened from the PI pool of patients; referral and/or from direct clinical visits. If the patient satisfies the eligibility criteria, he/she will be enrolled in the hematology clinic after signing the informed consent form.

### 8.3 Confidentiality

Patients’ data will be coded and kept in a secure database with unique username and password in order to maintain patient confidentiality. Only authorized research team will be granted access to the patients’ electronic charts and reports.

### 8.4 Access to data

Data collected in this study will be transferred to a secure database managed by the HMC IT team (eg, PACS). All data generated in this study will be the property of HMC. Original data acquired on the PET/CT scanner console will be destroyed as per the PET/CT center policy and procedures regulations.

### 8.5 Dissemination policy

The results and outcome of this study will be published in international medical journal and presented in international conferences for hematology radiology, hematopathology, and nuclear medicine.

## Author contributions

**Conceptualization:** Mohamed A. Yassin.

**Funding acquisition:** Mohamed A. Yassin.

**Methodology:** Mohamed A. Yassin, Sadek A. Nehmeh, Abdulqadir J. Nashwan, Samah A. Kohla, Shehab F. Mohamed, Omar M. Ismail, Ahmad Al Sabbagh, Firyal Ibrahim, Dina S. Soliman, Lajos Szabados, Hadi Fayad.

**Project administration:** Mohamed A. Yassin, Abdulqadir J. Nashwan.

**Writing – original draft:** Mohamed A. Yassin, Abdulqadir J. Nashwan, Samah A. Kohla, Shehab F. Mohamed, Omar M. Ismail, Ahmad Al Sabbagh, Firyal Ibrahim, Dina S. Soliman, Lajos Szabados, Hadi Fayad.

**Writing – review & editing:** Mohamed A. Yassin, Sadek A. Nehmeh, Abdulqadir J. Nashwan, Samah A. Kohla, Shehab F. Mohamed, Omar M. Ismail, Ahmad Al Sabbagh, Firyal Ibrahim, Dina S. Soliman, Lajos Szabados, Hadi Fayad.
